# From Notochord Formation to Hereditary Chordoma: The Many Roles of *Brachyury*

**DOI:** 10.1155/2013/826435

**Published:** 2013-03-31

**Authors:** Yutaka Nibu, Diana S. José-Edwards, Anna Di Gregorio

**Affiliations:** Department of Cell and Developmental Biology, Weill Medical College of Cornell University, New York, NY 10065, USA

## Abstract

Chordoma is a rare, but often malignant, bone cancer that preferentially affects the axial skeleton and the skull base. These tumors are both sporadic and hereditary and appear to occur more frequently after the fourth decade of life; however, modern technologies have increased the detection of pediatric chordomas. Chordomas originate from remnants of the notochord, the main embryonic axial structure that precedes the backbone, and share with notochord cells both histological features and the expression of characteristic genes. One such gene is *Brachyury*, which encodes for a sequence-specific transcription factor. Known for decades as a main regulator of notochord formation, *Brachyury* has recently gained interest as a biomarker and causative agent of chordoma, and therefore as a promising therapeutic target. Here, we review the main characteristics of chordoma, the molecular markers, and the clinical approaches currently available for the early detection and possible treatment of this cancer. In particular, we report on the current knowledge of the role of *Brachyury* and of its possible mechanisms of action in both notochord formation and chordoma etiogenesis.

## 1. Chordoma: Epidemiology, Classification, and Histopathological Characteristics 

Chordomas are very rare tumors that affect roughly one in a million individuals; the incidence in the US is ~300 new cases per year [1]. Yet, this rare neoplasm represents up to 4% of primary malignant bone tumors [2] and 20% of primary spine tumors [3]. 

Chordomas are classified on the basis of their location along the spine and their histological type. Depending on their location, chordomas are predominantly subdivided into clival (or skull-base), sacrococcygeal, cervical, thoracic, and lumbar. Even though historically the sacrococcygeal region was regarded as the most frequently occurring site for the formation of these tumors (e.g., [2]), recent studies have shown their almost equal distribution in the skull base, mobile spine, and sacrum [1]. 

Chordomas rarely occur in people below 40 years old; however, numerous cases of pediatric chordomas have been reported (e.g., [4, 5]) and generally were associated with cranial locations [6]. While cranially located chordomas affect both genders equally, sacrococcygeal tumors are more frequent in males, with the male : female ratio being approximately 2 : 1 ([7], and references therein). African-American individuals have been reported to be less frequently affected by chordoma [1], while Hispanic patients were found to have a significantly higher survival rate [6]. 

In addition to the more frequent axial tumors, extra-axial chordomas have also been reported. The location of extra-axial chordomas ranges from wrist [8] to feet [9]. These tumors have traditionally been identified through immunohistochemical studies [10, 11], and, more recently, through the use of the *Brachyury* gene as a novel diagnostic marker that distinguishes chordomas from similar lesions, such as myoepitheliomas and chondrosarcomas [9, 12]. 

Histologically, chordomas are categorized as classical (or conventional), chondroid, and dedifferentiated (e.g., [3]). The first microscopic characterization of chordomas dates back to 1857, when Virchow first identified the cells typical of this tumor and described them as “physaliferous” (Greek for “bubble-bearing”) because of the foamy appearance of their cytoplasm that contains multiple vacuoles [13]. Ultrastructural studies have indicated that the vacuoles can be divided into two subtypes, smooth-walled and villous, based upon the absence or presence of microvilli, respectively [14]. Physaliferous cells are typical of classical chordomas, appearing as groups of gray-white large cells separated by fibrous septa into lobules and surrounded by a basophilic extracellular matrix rich in mucin and glycogen [7, 15]. This is the most frequent type of chordoma. Its distinctive histological appearance led Müller to hypothesize, in 1858, that these tumors were of notochordal origin [16]; later, in 1894, Ribbert first introduced the term “ecchordosis physaliphora” [17], which is currently used to designate hamartomatous lesions of notochordal origin. Notochordal hamartomas are considered the benign counterparts of chordomas and are usually asymptomatic [18, 19]. While both ecchordosis physaliphora and chordoma are composed mainly of physaliphorous cells, stain for vimentin, the S-100 protein, epithelial membrane antigen, and low molecular weight cytokeratins, and are both negative for high molecular weight keratins [20], it is still unclear whether ecchordosis physaliphora can be a precursor of chordoma [19]; further investigations are needed to address this open question.

Chondroid chordomas show histological features resembling both chordoma and chondrosarcoma, a malignant tumor of the bone and soft tissue (e.g., [21]). This histological variant accounts for 5%–15% of all chordomas and up to 33% of all cranial chordomas, being preferentially found on the spheno-occipital side of the skull base [3]. Despite an appearance that resembles hyaline cartilage, these tumors retain an epithelial phenotype and express specific chordoma markers, including cytokeratin and S-100, which are not found in cartilaginous tissue; this has suggested their alternative, more appropriate classification as “hyalinized chordomas” [22]. Dedifferentiated chordomas are also rare, <10% of chordomas, and are characterized by sarcomatous regions, which are comprised of spindle-shaped polygonal cells (e.g., [23]). An important connection has been observed between the histological category of a chordoma and its ability to metastasize [24]: chondroid chordomas are the least aggressive, while dedifferentiated chordomas are the fastest-growing, more metastatic variety [3].

## 2. Therapeutic Approaches: Past and Present 

Since the late 1970s, treatment of chordoma has traditionally relied on *en bloc* surgical removal [25]; this technique has been very effective for sacral chordomas, although its applicability is obviously limited in the case of clival chordomas. Radical resection of the tumors and their capsules has proven to be considerably more effective than subtotal excision because it reduces the extent of cell seeding and, consequently, the risk of recurrence [26–28]. Advanced imaging techniques, including CT and MRI scans, allow for early detection of small intraosseous tumors and their complete resection [29, 30]. Since chordomas are radioresistant tumors, radiation therapy is usually employed in combination with surgery and mainly relies upon high-dose proton beam [31] and, to a more limited extent, on either helium- or carbon-ion therapy [32, 33].

Unfortunately, as slow-growing tumors, chordomas are resistant to conventional chemotherapies [34], and the use of alkylating agents, alkaloids, and related treatments has been reported to be modestly effective mostly on dedifferentiated chordomas [35]. However, increasing knowledge of the genetic markers of chordomas has led recently to the use of molecularly targeted therapies. The finding that chordomas display overexpression of constitutively active phosphorylated forms of platelet-derived growth factor receptor-(PDGFR)-b and of KIT receptors [36] prompted the use in chordoma patients of imatinib, a tyrosine-kinase inhibitor (TKI) drug with specificity for the kinase domains of these receptors. Imatinib was used with some success in clinical trials [37] alone or in combination with the immunosuppressant sirolimus (a.k.a. rapamycin) [38]. Another TKI, sunitinib, which targets multiple receptor tyrosine kinases and is used mainly for the treatment of gastrointestinal stromal tumors, has shown therapeutic efficacy against chordomas (e.g., [39]). It seems likely that the effects of TKI drugs may extend to other phosphorylated receptors found to be altered in chordomas, such as PDGFR-a [40], and to kinases that participate in additional pathways, such as Akt and mTOR, whose targets, TSC2 and the translation initiation factor binding protein EIF4EBP1, are found in a phosphorylated state in chordoma cells [41]. Other signaling pathways distinctive of chordomas that have been targeted are those linked to the epidermal growth factor receptor (EGFR); inhibitor drugs, such as cetuximab, gefitinib, and erlotinib, have been used in a small number of patients [42, 43]. The use of 9-nitro-camptothecin (9-NC), an inhibitor of topoisomerase I, was also limited to a small group of patients and seemed to delay cancer progression [44].

The development of chordoma cell lines in the past decade has also greatly widened the knowledge of the chordoma-specific molecular profile and has accelerated the identification of novel therapeutic targets. The first human chordoma cell line, U-CH1, was developed in 2001 [45] and has been used recently to test the efficiency of the various treatments described above [46]. Following the establishment of U-CH1, other cell lines were developed, including CH22 [47] and a cell line derived from a rare extra-axial chordoma, originally located in the scapula [48]. Other chordoma cell lines, CH 8 and GP 60, have also been generated successfully and tested for their ability to grow in different media [49]. Studies on these cell lines have identified new potential therapeutic agents, such as inhibitors of the transcription factor STAT3 [49]. In addition, work in chordoma cell lines, and parallel studies of familial chordomas, have revealed the role of another transcription factor, Brachyury, in the formation of this tumor. Short-hairpin RNA (shRNA)-mediated silencing of *Brachyury* in the cell line JHC7, derived from primary sacral chordoma, has shown that the downregulation of this transcriptional regulator leads to growth arrest [50]. Chromosomal gain at the *Brachyury * locus, seen in the MUG-Chor1 cell line, is found in a subset of familial chordomas [51] and is described in detail below.

## 3. Genetic and Cytogenetic Hallmarks of Chordoma

As described above, chordomas were classically identified on the basis of their histological features and their immunoreactivity for S-100 and markers such as epithelial membrane antigen (MUC1) and cytokeratins. However, the immunoreactivity for S-100, which is shared among chordomas and chondrosarcomas, made the assignment of tumors to either category quite challenging. This was particularly true when only small biopsies could be obtained, as in the case of clival chordomas (e.g., [52]). Another genetic marker of chordomas is chondroitin sulfate proteoglycan 4 (CSPG4), which has also been targeted for immunotherapy; however, this antigen is expressed only by a fraction (~62%) of these tumors [53] and could therefore produce false negative diagnoses. These limitations prompted a widening of the search for distinctive chordoma markers to transcription factors. The Sry-type HMG-box transcription factor Sox9, which is required for the maintenance of notochord integrity [54], was found to be expressed in both kinds of tumors [55]. Conversely, the detection of Brachyury, a member of the T-box family, used in combination with cytokeratin, provided a considerably accurate distinction of chordomas from chondrosarcomas [55]. Further studies revealed specific expression of *Brachyury* in >90% of the chordomas analyzed, and validated its use as a specific marker of this tumor [52, 53, 56]. Nevertheless, it is noteworthy that *Brachyury* is overexpressed in a number of tumor types, including lung cancer, and is a potent mediator of epithelial-mesenchymal transition (EMT) [57, 58], one of the main mechanisms responsible for metastasis (e.g., [59]).

Numerous cytogenetic abnormalities have been reported in chordomas and have been used to aid in their classification, the most common being the partial or complete loss of chromosome arms 1p and 3p, and the partial or complete gain of chromosomes 7 and 20 [60]. In particular, chromosomal regions 1p36 (the *RIZ* locus, encoding a zinc-finger protein; [61]), 1q25 (encompassing the hereditary human prostate cancer susceptibility locus *HPC1*, [62]), 2p13 (the *TGF*-*alpha * locus), and 7q33 (the *AKR1B10* locus, encoding an aldo-keto reductase; [63]) display either deletions or amplifications in primary chordomas [64]. Other frequent deletions reported in chordoma occur in the *CDKN2A* and *CDKN2B* loci, both on chromosome 9p21, which encode the cyclin-dependent kinase inhibitors known as p16 and p15, respectively [65]. Of note, chordomas are among the cancers that display a newly described mechanism of chromosomal disruption, called chromothripsis, which is found in ~25% of bone cancers and involves the massive fragmentation of one or more chromosomes, followed by their imperfect reassembly [66].

## 4. Duplications and Mutations of *Brachyury* in Hereditary and Sporadic Chordomas

Some of the most relevant advancements in the search for the molecular bases of chordoma are the result of systematic studies of families affected with this cancer. The first of these analyses showed genetic linkage to chromosome 7q33 [63], which was corroborated by later research [67]; however, an additional study on the same group of families revealed that one of the individuals affected by chordoma had not inherited the 7q33 haplotype, thus prompting the search for additional susceptibility loci [68]. In turn, these efforts led to the identification of a 6q haplotype shared by all affected individuals in one of the families and defined a minimal disease region spanning ~5 Mb [68]. Through the analysis of copy number variants (CNVs), this interval was found to contain regions of 6q27 duplicated to various extents. Eventually, the duplicated areas shared by all families were narrowed to an ~100 kb interval that contains only one locus, *Brachyury* [68, 69].

Subsequent work analyzed the frequency of *Brachyury* amplifications in 181 sporadic chordoma samples: 7% of the tumors displayed amplification of the *Brachyury* locus, 39% of the tumors were polysomic for chromosome 6, and 4.5% of primary tumors showed a minor allelic gain of *Brachyury* [70]. No relationship was observed between the frequency of amplifications in these sporadic chordomas and the region of origin of the tumors [70]. Of note, no germline alteration of the *Brachyury* locus was identified in non-neoplastic tissue from 40 patients, indicating that the copy number gain is somatic [70].

In sum, these studies demonstrated that chromosomal aberrations resulting in copy number gain of *Brachyury* are found in both sporadic [70] and hereditary chordomas [68]. Consistently, the expression of this gene is critical for the proliferation of chordoma cell lines *in vitro*, as shown by the results of the silencing experiments reported above [50]. Furthermore, the combination of traditional Sanger sequencing with recently developed whole-exome sequencing methodologies allowed an extensive genotyping study of germline DNA from 45 individuals with sporadic chordoma, focused on the *Brachyury* coding region [71]. The results revealed a recurring single nucleotide polymorphism (SNP), rs2305089 that lies in exon 4, which encodes part of the DNA-binding domain of Brachyury [71]. This mutation, therefore, alters the DNA-binding properties of this transcription factor, suggesting that the misregulation of some of the genes controlled by Brachyury could be a mechanism underlying the genesis of chordoma. Brachyury-downstream genes identified so far in various model organisms are discussed in detail in a later section. 

## 5. The Role of *Brachyury* in Notochord Evolution and Development

Long before its identification as a marker and a possible cause of chordoma, *Brachyury* was already well known as a major regulator of notochord formation. The notochord is the main defining feature of the chordate phylum, which includes humans and all other vertebrates. This embryonic structure provides crucial support to the developing embryo, patterns the central nervous system, and ensures the proper development of a variety of organs, from heart and aorta to liver and pancreas [72]. As the ossification of the spine proceeds and vertebral bodies form, the notochord regresses and eventually its remnants become the *nuclei pulposi* (NP) of the intervertebral discs. The histological identity of these cells remained controversial, since cells of the NP were described as either “chordoid,” that is, notochordal, or as “chondroid,” that is, as chondrocytes [73]. It has recently been clarified, through lineage-tracing experiments, that notochord cells are the embryonic precursors to all NP cells and to the mature intervertebral discs [74]. 

The *Brachyury* (Greek for “short tail”) mutation was first identified in 1927 by Nadine Dobrovolskaïa-Zavadskaïa through the observation of short-tailed mice, which were later found to be heterozygous for a mutation in the eponymous locus. Subsequent studies confirmed that the mutation was dominant, attributable to a single factor, and accompanied by abnormalities of the posterior skeleton [75], particularly in the number of presacral vertebrae [76]. It was also determined that homozygous *Brachyury* embryos die *in utero* by gestation day 11 due to severe defects in the formation of the allantois [77]. Interestingly, these embryos fail to form a notochord and, as a consequence, show severe abnormalities in the development of the neural tube and somites [78]. 

The identification of the gene* Brachyury* (or “*T*,” for “tail”), which in mouse is part of the *t*-complex that spans 40 cM on chromosome 17 [79], did not occur until 1990; the gene was isolated by positional cloning [80] and found to be expressed in notochord and primitive-streak mesodermal cells [81]. Three years later, the Brachyury protein was classified as a novel sequence-specific transcription factor [82]. Parallel studies identified *Brachyury* orthologs in *Xenopus*, zebrafish and chick [83–86]. Of note, the immature NP cells of the intervertebral discs were found to express both Brachyury mRNA and protein [56, 87].

The discovery of *Brachyury* orthologs in basal chordates, such as the ascidians *Halocynthia* and *Ciona* [88, 89] and the amphioxus *Branchiostoma* [90], and the remarkable conservation of their function underscored the paramount role played by this developmental regulator in the appearance of the notochord, the main event in the evolutionary history of the phylum Chordata. However, the appearance of *Brachyury* genes predates that of the notochord by several million years. In fact, although *Brachyury* orthologs have not been found in plants, genes related to *Brachyury* have been identified recently in the fungus *Spizellomyces punctatus* and in the amoeba *Capsaspora owczarzaki* [91], shifting the long-standing paradigm that considered *Brachyury* and its related genes as metazoan-specific innovations. Rather, it has become evident that the expansion of the repertoire of genes encoding transcription factors related to Brachyury accompanied the evolution of multicellularity in the animal kingdom. Indeed, Brachyury is the founding member of a family of transcription factors that share related DNA-binding domains and are therefore collectively designated as T-box (Tbx) proteins [92]. The availability of full genomic sequences for numerous animal species has confirmed the nearly ubiquitous representation of *Brachyury* and other *Tbx* genes throughout different phyla. For example, single-copy *Brachyury* and *Tbx2/3* orthologs have been reported in the placozoan *Trichoplax adhaerens* [93] and *Brachyury* and other *Tbx* genes have been described in sponges (e.g., [94]). Likewise, *Brachyury* and *Tbx2/3* orthologs have been reported in *Pleurobrachia pileus*, a member of Ctenophora (comb jellies), one of the first metazoan phyla [95].

Placozoan *Brachyury* is expressed in scattered individual cells that do not coincide with known anatomical structures [93], and its function in this simple organism is still unknown. However, several lines of evidence suggest that the ancestral function of Brachyury was the regulation of morphogenetic movements [96]. Consistently, in the sponge *Suberites domuncula*, *Brachyury* is expressed at the time when cell-cell and cell-matrix interactions are being established, suggesting that it might regulate cell adhesion and migration. The ctenophore *Mnemiopsis leidyi *expressed *Brachyury* (*MlBra*) in ectodermal cells around the site of gastrulation as well as in cells deriving from the blastopore; morpholino oligonucleotide-mediated knockdown supports its involvement in gastrulation movements [97]. In the sea anemone *Nematostella vectensis*, a basal cnidarian, expression of *Brachyury* is restricted to a circle of cells surrounding the blastopore [98]. 

In protostomes, a large division of animals including arthropods, mollusks, annelids, and other less-known taxa, the blastopore is the embryonic region that gives rise to the mouth. *Drosophila Brachyury,* called *brachyenteron*, is required for the formation of the hindgut and of the midgut constrictions, and for the elongation of Malpighian tubules [99]. This function in the formation of posterior structures is conserved in other arthropods, including the beetle *Tribolium* and the grasshopper *Locusta* [100]. Notably, Brachyenteron synergizes with the winged-helix transcription factor Forkhead to specify the caudal visceral mesoderm of *Drosophila*, a remarkable parallelism with the roles played in mesoderm formation by chordate Brachyury and Forkhead (Fox) orthologs [101, 102].

Lastly, *Brachyury* orthologs have also been studied in the two main phyla of non-chordate deuterostome animals, echinoderms (e.g., sea urchins and sea stars) and hemichordates (e.g., acorn worms). In the sea urchin *Lytechinus variegatus*, microinjection of a dominant-negative form of its *Brachyury* ortholog caused a block in gastrulation [103]. While there are as yet no functional studies of *Brachyury* in hemichordates, it has been shown that in the acorn worm *Ptychodera flava*, *Brachyury* is expressed in the blastopore at the time of gastrulation, and later in the stomodeum, the precursor of the mouth and anterior pituitary gland [104].

Together, these functional analyses in different representative organisms along the phylogenetic tree indicate that the ancestral function of *Brachyury* was the promotion of cell movement and adhesion, which are fundamental for both morphogenesis and tumorigenesis. As the evolution of multicellular organisms proceeded, this gene became involved in the specification of an area of the blastopore with distinctive properties in axis formation, and later on turned into a driver of mesoderm specification (e.g., [105]). Eventually, possibly through the synergistic interaction with other transcription factors, such as members of the Fox family, Brachyury acquired its crucial role in notochord formation.

## 6. Possible Mechanisms of Action of Brachyury during Tumorigenesis: The Brachyury-Downstream Gene Battery

Brachyury has been reported to act as a transcriptional activator, although it possesses two repression domains in its C-terminal region [106]. Crystallographic studies elucidated the conformation of the DNA-binding domain (T-domain) of *Xenopus* Brachyury (Xbra) bound to a 24-nucleotide palindrome and revealed the peculiar interactions between the C-terminus of the Xbra protein and the minor groove of DNA [107]. Subsequent studies using electrophoretic mobility shift assays (EMSA) have shown that Xbra and other Brachyury orthologs can also bind sequences that correspond to roughly half of the palindrome originally identified by Kispert and Herrmann [82]. In fact, numerous functional non-palindromic binding sites, also designated as “half-sites,” with the minimal generic consensus sequence TNNCAC [108], have been identified in different model systems (e.g., [109–111]). A longer, non-palindromic consensus binding site, RWWNTNRCACYT, was also identified for *Drosophila* Brachyenteron based on its interaction with the *orthopedia* gene [112]. The *orthopedia* regulatory region contains multiple copies of this site, which are bound cooperatively by Brachyenteron and are also recognized *in vitro* by mouse and *Xenopus* Brachyury [112].

As the knowledge of Brachyury binding preferences continued to accumulate, the quest to identify its downstream genes gained momentum and led to the discovery of numerous Brachyury targets in chordates, but also in nonchordate model systems, such as the sea urchin [113]. In chordates, Brachyury targets were first identified in *Xenopus* and the invertebrate chordate *Ciona intestinalis* and, more recently, in zebrafish, mouse stem cells, and chordoma. 

Studies in *Xenopus* led to the identification of five putative targets of Xbra: the signaling molecule Xwnt11, the zinc-finger transcription factor Xegr-1, a member of the BTG/Tob family of antiproliferative proteins, Xbtg1, a protein of unknown function, BIG3/1A11, and the homeodomain transcription factor Bix1 [114, 115]. Follow-up studies on Xwnt11 indicated that Xbra controls gastrulation movements through this signaling molecule [116]. Additional work revealed that Xbra binds the regulatory region of *eFGF* to regulate its expression [109, 117]. Together, these results began to delineate the gene regulatory network controlled by Brachyury, first during gastrulation and later in mesoderm formation. 

The identity of numerous notochord-specific targets of Brachyury was later revealed by studies in *Ciona intestinalis*, since in this and other tunicates the expression of *Brachyury* (*Ci-Bra*) is confined to the notochord [89]. A subtractive screen aimed at identifying Ci-Bra-downstream genes first uncovered ~40 genes expressed in notochord cells [110, 118–121]. Interestingly, in contrast to the earlier results in *Xenopus*, most of the validated notochord Ci-Bra targets encode various structural proteins, including a non-muscle tropomyosin, fibrillar collagen, laminins, and thrombospondin [110, 122, 123]; however, components of the Wnt/planar cell polarity pathway, such as Prickle, and mediators of TGF-beta and NF-kappaB signaling were also discovered [121]. Subsequent studies expanded the number of possible components of the Ci-Bra-downstream regulatory hierarchy [119, 122] and recent genome-wide chromatin occupancy studies (ChIP-chip assays) suggested that the number of loci directly bound by Ci-Bra in early *Ciona* embryos is close to 2,000 [124]. In addition, notochord transcription factors of the STAT, Fos, AFF, Krüppel-like (Klf), and NFAT5 families were recently indicated as Ci-Bra targets, suggesting that the gene regulatory network controlling formation of the notochord in this simple chordate is multitiered, and that these transcription factors might have been reiteratively recruited for notochord development by other chordates [125]. Of note, in vertebrates *NFAT5* was found to be expressed in the NP, where it is required for the survival of the notochord cells in response to hyperosmotic stress [126]. 

In zebrafish, a *Brachyury* ortholog, no tail (ntl), has been found to control the expression of numerous target genes, including transcription factors as well as genes that participate in signaling and differentiation pathways [127]. These genes are involved in a variety of cellular processes, including morphogenetic movements (e.g., *wnt11*, *snail1a*, *connexin 43.3,* and *tbx16*), muscle specification (e.g., *mesogenin1* and *myoD*), and generation of posterior identity (e.g., *tbx6*, *vent*, *vox*, *fgfr4,* and *cdx *genes) [127]. Among the Ntl target genes is also *floating head*, the ortholog of the mammalian homeobox transcription factor gene *Noto*, which is required for the formation of the posterior notochord [128]. 

Putative target genes of mouse Brachyury, 396 in total, have been recently identified in mouse differentiating stem cells [129]. Similarly to zebrafish Ntl, mouse Brachyury controls several transcription factors of the homeobox, winged-helix, paired box, zinc-finger, and odd-paired families; it also regulates components of the Wnt signaling pathway, the growth factor Fgf8, cytoplasmic dyneins, and orthologs of the transcriptional repressor Snail [129]. However, this study also identified Brachyury targets that were not identified by the zebrafish study: Axin2, a negative regulator of the Wnt pathway, gamma-catenin/Jup, Fgf8, and Wnt3A; interestingly, ChIP-qPCR experiments showed that these genes were also bound by human Brachyury in hESC-derived mesoderm cells [129]. 

It is noteworthy that one of the recurring targets of Brachyury in different animals, including *Ciona*, zebrafish and mouse, is the gene *Snail * [124, 127, 129]. *Snail * encodes a transcriptional repressor that, along with transcription factors of the ZEB, Slug, and Twist families, is a mediator of EMT [130, 131]. This suggests that in tumor cells Brachyury might induce EMT through activation of Snail and its downstream genes. Another conserved regulatory interaction occurs between Brachyury and FGF factors, as demonstrated by the Brachyury/FGF autoregulatory loops seen in *Xenopus* and zebrafish [109, 132, 133] and by the binding of human Brachyury to the *FGF8* promoter region [129]. A link between FGF and Brachyury has also been found in ascidians; in *H. roretzi*, the FGF/MEK/MAPK/Ets signaling pathway is required for the initial expression of *Hr-Bra* through the binding of Ets to the 5′-flanking region of this gene [134]. Similarly, in early *Ciona* embryos, FGF9/16/20 promotes the activation of *Ci-Bra* expression, while subsequently FGF8/17/18, together with FGF9/16/20, ensures its maintenance [135].

The identity of Brachyury target genes in humans has been recently revealed by transcriptome and ChIP-Seq analyses in the chordoma cell line U-CH1 [136]. This study revealed that a large subset of Brachyury targets is involved in cell cycle control, including NUSAP1 and BUB1, spindle checkpoint genes involved in cell proliferation [137, 138]. Another major division of chordoma Brachyury targets is represented by genes encoding extracellular matrix (ECM) components, including *laminin alpha2*, *collagen type VI alpha3,* and *olfactomedin 4 *[136]. Lastly, other Brachyury targets of potential medical relevance are chemokines and growth factors, including connective tissue growth factor (CTGF), which has also been reported to control motility and signaling in the notochord [139]. 

Somewhat surprisingly, a preliminary comparison of the Brachyury targets identified in mouse differentiating stem cells with those found in chordoma does not reveal numerous commonalities. Among the shared Brachyury targets are the Ets-family transcription factor ETV1, which is translocated in certain sarcomas [140], CIT (citron Rho-interacting kinase; [141]), GABRA2 (GABA receptor alpha2), the zinc-finger protein ZDHHC17, which has been previously found to be upregulated in leukemic cells over-expressing Gfi-1B [142], PIGK (phosphatidylinositol glycan, class K), which was found to be downregulated in colorectal cancer due to a polymorphism in its 3′-UTR [143], TNFRSF19 (tumor necrosis factor receptor superfamily, member 19, a.k.a. TROY), previously found to be over-expressed in glial tumors [144], VEPH1, encoding a PH-domain protein expressed in the developing central nervous system [145], PTN (pleiotrophin), which is expressed in the canine notochord and NP [146], SCRG1, encoding an ECM-localized protein involved in chondrogenesis [147], and the cell proliferation activator TGF-alpha, which has been shown to stimulate proliferation of chondrocytes [148]. Interestingly, with the exception of PTN, none of the other genes was previously reported as a possible notochord marker, which likely reflects the fact that cancerous hallmarks are chordoma-specific targets of Brachyury.

## 7. Possible Mechanisms of Action of Brachyury during Tumorigenesis: Interaction with Other Factors 

Brachyury proteins can homodimerize when contacting DNA and bind either palindromic sequences or inverted repeats in this form [82, 106, 107]; in addition, Brachyury can also bind DNA and activate transcription as a monomer [108–110]. The homodimerization of mouse Brachyury is mediated by a short peptide, PESPNF, which was also found to allow its interaction *in vitro* with Brachyury proteins from other species, such as *Drosophila* Brachyenteron [112]. These findings have led to the hypothesis that Brachyury might be able to form heterodimers with other T-box proteins [149]. 

In terms of non-T-box proteins, Brachyury has been shown to interact with Smad1, a component of the BMP signaling pathway, through a short N-terminal peptide; this region is found only in Brachyury proteins from bilaterian animals [150, 151]. The interaction with Smad1 was shown to restrict the inductive ability of the bilaterian Brachyury proteins to mesoderm formation in *Xenopus* animal cap assays [150]. While the evolutionary significance of this interaction has been clarified, it is still unknown whether this and/or additional protein-protein interactions might also modulate the oncogenic properties of Brachyury. 

Another protein that interacts directly with Brachyury is the homeodomain transcription factor Mixl1. *In vitro* studies revealed that these proteins were associated *via* their respective DNA-binding domains [152]. In particular, the T-domain, contained within the first 230 amino acids of the N-terminal region of Brachyury, interacts with helix III of the Mixl1 homeodomain, preferentially when Mixl1 is in its homodimeric form [152]. This interaction with Brachyury does not impede Mixl1 binding to DNA, suggesting that Brachyury might be recruited to DNA by DNA-bound Mixl1 and, thus, regulates some of its target genes without directly binding DNA [152]. Interestingly, the same study proposes that the interaction of the Brachyury N-terminal region with Mixl1 might expose the C-terminal repression domains of Brachyury, leaving them available for interaction with transcriptional corepressors. A similar model has been brought forth in a study of the Ripply proteins, which act as adaptors and recruit the corepressor Groucho/TLE to T-box proteins, shifting their transcriptional activity towards repression [153]. Although a direct interaction with Ripply1 has so far only been shown in the case of Tbx24, another T-box transcription factor related to Brachyury, it is possible that a similar interaction might occur with Brachyury as well. Indeed, Ripply1 is able to suppress the activating ability of Ntl in zebrafish [153].

Even though a physical interaction between Brachyury and members of the Fox subfamily of winged-helix transcription factors has not been reported, it is noteworthy that these transcription factors have been found to work synergistically in different model systems. For example, in *Xenopus*, animal caps from embryos coinjected with *pintallavis*, a member of the Foxa4 class, and *Xbra* mRNAs give rise to mesoderm and notochord [154]. In *Drosophila*, *brachyenteron* (*byn*) and *forkhead* (*fkh*) cooperatively specify the caudal visceral mesoderm, which is absent in *byn*/*fkh* double mutant flies and is formed ectopically only when both genes are over-expressed [101]. In the mouse embryo, both *Brachyury* and *Foxa2 * have been reported to be upstream of the homeobox gene *Noto*, based upon the evidence that this gene is down-regulated in mutant embryos for either gene [155]. Finally, studies in ascidians have identified a compact notochord *cis*-regulatory module whose activity depends on both Ci-Bra and Ci-FoxA2 binding sites, thus providing the first report of a synergistic interaction between these factors within a compact regulatory region [156].

Together these studies suggest that the various interactions of Brachyury with other proteins exert a considerable influence on its function, suggesting that their impairment could be both a cause and a consequence of tumorigenesis.

## 8. Possible Mechanisms of Action of Brachyury during Tumorigenesis: Dosage-Dependent Effects

We have previously discussed the duplication of the *Brachyury* locus as a hallmark of hereditary chordoma. It is important to note, however, that duplications of *Brachyury* have occurred in various species along the phylogenetic tree without negative consequences. For example, two *Brachyury* genes have been identified in the cnidarian *Hydra vulgaris*. Both genes are expressed in the hypostome, although *HyBra1* is expressed predominantly in endoderm, and *HyBra2* in ectoderm [157]. In *Xenopus* animal cap assays, only *HyBra1* is capable of inducing mesoderm formation, similarly to vertebrate Brachyury proteins [157]. In basal chordates, there is only one copy of *Brachyury* in *Ciona* and other tunicates [89, 135], while the cephalochordate amphioxus has two copies, likely the result of a lineage-specific duplication [90]; both amphioxus genes are expressed in the mesoderm and subsequently in the differentiating notochord [158]. In vertebrates, two *Brachyury* orthologs have been found in zebrafish, the well-characterized *no tail*, and its paralog *Brachyury *(recently renamed *ntl-a* and *ntl-b*, respectively); combined inactivation of these genes exacerbates the *ntl-a* mesodermal phenotype, indicating that these factors act synergistically [159]. Also, in mouse, another *Brachyury* gene, *Brachyury the Second* (*T2*), has been found and mapped to the proximal region of chromosome 17 near *Brachyury*; a mutation in this gene reportedly causes defects in notochord formation, although not as severe as those seen in *Brachyury* mutants [160]. *T2* does not seem to be present in the human genome.

Therefore, it appears that duplicated copies of *Brachyury* genes can become functional paralogs, and either segregate into different embryonic territories or continue to be expressed within the same tissue; in the latter case, they may act synergistically and likely control shared target genes. Nevertheless, this partial or complete functional redundancy is expected to require strict tissue-specific regulation in order to prevent the damage that an excessive level of Brachyury protein could cause. In fact, the increased dosage of Brachyury has been shown to exert strong effects on development, as seen in transgenic mouse embryos where three copies of this gene resulted in an extension of the anterior-posterior axis [161]. The main molecular mechanism underlying this effect is likely represented by the dose-dependent binding of Brachyury to the *cis*-regulatory regions of its target genes. An example of this mechanism has been documented in the case of the *Drosophila* gene *orthopedia* that, as mentioned above, is controlled in a dose-dependent fashion by Brachyenteron through multiple binding sites [112]. It has recently been suggested that this mode of gene regulation, whereby Brachyury proteins utilize modular arrangements of canonical and noncanonical binding sites to control some of their targets, might be employed by mouse Brachyury as well [129]. In conclusion, the levels of functional Brachyury protein modulate the function of this transcription factor, most likely by altering the regulation of its direct target genes.

## 9. Possible Mechanisms of Action of Brachyury during Tumorigenesis: Upstream Regulators of *Brachyury* Transcription

While the identities of Brachyury-downstream genes continue to be elucidated, in parallel, several groups have studied the regulation of the expression of *Brachyury* itself using different model systems. The emerging consensus is that the essential signaling pathways that govern body plan formation and mesoderm induction, Wnt/*β*-catenin, TGF-*β*/Nodal/activin, BMP, and FGF, also act upstream of *Brachyury* in concert with different combinations of early-onset transcription factors. The involvement and cross-interactions of each of these pathways vary among different animals. In zebrafish, a Nodal morphogen gradient activates expression of both *Brachyury* orthologs, *ntl-a* and *ntl-b*; however, transcription of *ntl-a* is additionally regulated by both Wnt and BMP signaling, while transcription of *ntl-b* is not responsive to these pathways [162]. The BMP signaling pathway and zygotic Wnt are also upstream of *Xbra* in *Xenopus* [163, 164]. In humans, BMP-4 has been indicated as a regulator of *Brachyury* transcription, being able to induce its expression in hES cells; the same study showed that activin-A and Wnt3a also exhibited minor inducing ability [165].

In some instances, differences in the regulation of *Brachyury* expression are observed between different regions of the notochord in the same animal. For example, in the ascidian *Ciona*, the first step leading to the activation of *Brachyury* expression in the main notochord lineage (the 32 anterior notochord cells, or A-lineage) is the translocation of cytoplasmic *β*-catenin to the nuclei in vegetal blastomeres, an event that activates transcription of *FGF9/16/20* and of the transcription factor FoxD [166]. In turn, FoxD activates expression of the zinc-finger transcription factor ZicL that, along with FoxD, binds the *Brachyury* notochord CRM and activates transcription [167]. However, in the posterior-most 8 notochord cells (secondary notochord, or B-lineage), *Brachyury* expression is instead regulated by Nodal and Delta-like [168]. 

Although the signaling pathways upstream of *Brachyury* overall appear to be conserved across the phylogenetic tree, the transcription factors that control expression of this gene vary among different organisms. In *Ciona*, Ci-FoxA2 has also been reported to activate *Brachyury* expression [169], and this might be the case in other organisms where the expression of *Foxa2* precedes that of *Brachyury* genes. The finding that the mouse *Foxa2* promoter region is bound by Brachyury suggests that the two genes might be regulating each other [129]. In addition to mesoderm inducers, a factor mainly known for its involvement in innate and adaptive immune response, NF-kappaB, has been shown to modulate the transcription of *ntl-a* in zebrafish and of *Xbra* in *Xenopus* [170]. Furthermore, it seems reasonable to hypothesize that after their transcription is initiated, by one or more of the aforementioned pathways, Brachyury proteins can positively regulate their own transcription, either directly or indirectly, as is the case for other transcription factors. In fact, autoregulation has been reported in the case of Xbra [171], and ChIP-Seq experiments have shown that human Brachyury binds its own locus [136], although the existence of this autoregulatory loop has been ruled out in mice [172]. 

Transcription of *Brachyury* is also modulated by repressive interactions. In *Ciona*, expression of *Ci-Bra* is excluded from muscle cells, and thus confined to the notochord, by the zinc-finger transcriptional repressor Snail [173]; in *Xenopus*, *Xbra* is actively silenced by another zinc-finger repressor, Smad-interacting protein (SIP1) [174]. Also in *Xenopus*, Xbra and the homeodomain protein goosecoid repress each other, while transcription of both genes is stimulated by activin [175]. This interaction is evolutionarily conserved, as shown by the finding that *Brachyury* is repressed in goosecoid-overexpressing mouse ES cells [176]. Repression can also take place indirectly, as it is seen in the case of embryonic stem cells, where the Smek/PP4c/HDAC1 complex binds the Tcf/Lef binding site of the *Brachyury* promoter and causes histone deacetylation, thus impeding transcription [177].

## 10. Concluding Remarks

From the studies that we have summarized above, it seems that Brachyury-expressing cells have the potential to somehow give rise to chordomas, possibly through one or more of the mechanisms that we have outlined. If this is the case, it remains to be determined whether and how normal Brachyury-expressing notochord cells turn into the Brachyury-expressing cells found in chordomas. Possible clues to the solution of this problem might come from a recent gene expression study showing that chordomas contain cancer stem-like cells [178]. In this study, some of the chordoma cells from the U-CH1 cell line were found to express stem cell surface markers. These results provide the first evidence that chordomas might contain cancer stem cells (CSCs) and could open new avenues for research on possible treatments. Intriguingly, recent studies carried out in adenoid cystic carcinoma cell lines indicate that CSC characteristics can be reversed by the knockdown of *Brachyury*, along with the ability of these cells to undergo EMT [179]. Taken together, these recent findings again point at *Brachyury *as a possible therapeutic target. Within this context, the discovery of the immunogenic properties of Brachyury and the subsequent development of specific CD8^+^ cytotoxic T-cells able to destroy Brachyury-expressing tumor cells from various cells lines *in vitro* [130, 180] represent an encouraging step towards the development of Brachyury-targeted therapies. More generally, therapeutic agents targeting Brachyury could also be employed for cancer immunization strategies directed against any cancer cell undergoing EMT that expresses this marker [130]. Brachyury-targeted therapeutics could possibly be implemented through adaptations of recently developed exosome-based nanodevices, which increase the immunogenicity of tumor-associated antigens [181]. Lastly, new treatments could be directed against one or more of the numerous genes controlled by Brachyury, to increase their specificity.
